# OpenVigil FDA – Inspection of U.S. American Adverse Drug Events Pharmacovigilance Data and Novel Clinical Applications

**DOI:** 10.1371/journal.pone.0157753

**Published:** 2016-06-21

**Authors:** Ruwen Böhm, Leocadie von Hehn, Thomas Herdegen, Hans-Joachim Klein, Oliver Bruhn, Holger Petri, Jan Höcker

**Affiliations:** 1 Institute of Experimental and Clinical Pharmacology, University Hospital Schleswig-Holstein, Campus Kiel, Kiel, Germany; 2 Department of Computer Science, Christian-Albrechts University, Kiel, Germany; 3 Hospital pharmacy, Wicker Kliniken, Bad Wildungen-Reinhardshausen, Germany; 4 Department of Anaesthesiology and Intensive Care Medicine, University Hospital Schleswig-Holstein, Campus Kiel, Kiel, Germany; National Chiao Tung University, TAIWAN

## Abstract

Pharmacovigilance contributes to health care. However, direct access to the underlying data for academic institutions and individual physicians or pharmacists is intricate, and easily employable analysis modes for everyday clinical situations are missing. This underlines the need for a tool to bring pharmacovigilance to the clinics. To address these issues, we have developed OpenVigil FDA, a novel web-based pharmacovigilance analysis tool which uses the openFDA online interface of the Food and Drug Administration (FDA) to access U.S. American and international pharmacovigilance data from the Adverse Event Reporting System (AERS). OpenVigil FDA provides disproportionality analyses to (i) identify the drug most likely evoking a new adverse event, (ii) compare two drugs concerning their safety profile, (iii) check arbitrary combinations of two drugs for unknown drug-drug interactions and (iv) enhance the relevance of results by identifying confounding factors and eliminating them using background correction. We present examples for these applications and discuss the promises and limits of pharmacovigilance, openFDA and OpenVigil FDA. OpenVigil FDA is the first public available tool to apply pharmacovigilance findings directly to real-life clinical problems. OpenVigil FDA does not require special licenses or statistical programs.

## Introduction

Pharmacovigilance collects spontaneous reports or–nowadays–analyzes prescription data, social internet platforms or electronic health records in hospitals to gather information of drug usage and subsequent adverse events (AE) which could be attributed to the usage of a (new) drug, i.e., adverse drug reactions (ADR) [1, 2].

The U.S. American Food and Drug Administration (FDA) has recently began to establish a powerful way to access Adverse Events Reporting System (AERS) pharmacovigilance records from mid-2003 until to date: the openFDA Application Programming Interface (API) via internet using standard data transport methods and data formats (HTTP/JSON) [3].

One year after the initial beta release, implementations for the programming language Ruby and the statistical environment R as well as several web-based services are available (Table 1). However, analysis options are limited: the web-based services do not provide sufficient research options, whereas the library for data import in R requires a deep understanding of both openFDA and R by the user. This substantial gap is closed by OpenVigil FDA.

**Table 1:** Available openFDA front-ends.

Type of resource	Unified Resource Locator (URL) or name of software package	Author / Maintainer
web-based	https://openfda.shinyapps.io/RR_D	FDA Office of Informatics and Technology Innovation
	http://searchopenfda.socialhealthinsights.com/	Social Health Insights LLC
	http://genderedreactions.com/	The Think Train & Centre for Gender Medicine
	http://www.visualizefda.com/	Anonymous
others	rOpenHealth/openfda	Russell Power <power@iodine.com>
	open_fda for Ruby	Jason Blalock

The OpenVigil pharmacovigilance analysis project (http://openvigil.sourceforge.net) [4] provides intuitive user interfaces, powerful algorithms and highly configurable output of findings, both individual reports and counts, in various output formats. OpenVigil FDA is our latest web-based analysis tool which was designed to work with openFDA exclusively. Of note, there are other applications to mine pharmacovigilance data available (Table 2). However, they employ no or different data cleaning techniques than openFDA. Since openFDA is expected to complement and later replace the traditional mode of access to FDA AERS data, i.e., downloading raw data files, it is important to analyze its capabilities and limitations.

**Table 2:** Comparison of open-access web-resources that mine FDA Adverse Events data.

category	FDAble	DrugCite	ehealthme	OpenVigil 1	OpenVigil 2	OpenVigil FDA	AERS Spider	AERS*Mine*	CzeekV
Basic drug-ADRs risk detection (e.g., PRR)	+	-	-	+	+	+	+	+	?^5^
Drug entries normalized to generic names (includes brands, spelling variants, foreign names, typos etc.)	?^1^	?^1^	?^1^	-^2^	+	+	+	+	+
Ontological aggregation of drugs	-	+	-	-^2^	+	+	+	+	+
Ontological aggregation of indications	-	-	-	-^2^	-^3^	-	+	+	?^5^
Ontological aggregation of adverse events	-	+	-	-^2^	-^3^	-	+	+	+
Integration of known on-label ADR	-	-	+	-	-	+	-	+	?^5^
Simultaneous investigation of multiple drugs, indications and adverse events	-	-	-^4^	+	+	+	+	+	+
Detect confounders	-	-	-	-	-	+	+	+	?^5^
Mask confounders / Correct background	-	-	-	-	+	+	+	+	+
Analyse multiple cohorts	-	-	-	-	-	-	-	+	-
Import/export direct database queries (e.g., SQL)	-	-	-	+	+	+	-	-	-
Advanced drug-ADR risk detections (e.g., Bayes’)	-	-	-	-	+	-	-	+	?^5^
Automatic visualisation	-	-	-	-	-	+	-	+	?^5^
Other datasets than AERS (e.g., German)	-	-	-	+	+	-	-	-	-
Free to use	-^6^	+	+	+	+	+	+	+	+
Open-sourced and transparent data	-	-	-	+	+	+	-	-	-
Save queries/analyses	-	-	-	+	+	+	-	+	-

Table adapted from https://research.cchmc.org/aers/help/supplements/Supplementary%20Table%203%20-%20Comparison%20of%20web-resources%20that%20mine%20the%20FAERS%20data.pdf (Accessed 7 May 2016).

1: Information on the data cleaning procedures are not available due to missing documentation of the close-sourced engines

2: OpenVigil 1 does not contain any mapping logic but pre-sanitized and ontology-enriched data can be loaded

3: the official MedDRA ontology has been licensed and is being incorporated into OpenVigil 2.2

4: only pre-defined analyses scenarios

5: search engine and related literature are Japanese only and therefore, cannot be understood

6: only free to browse for the first results.

Generally, ontologies for drugs and events are being incorporated into all major search engines thus initiating a transition of simple drug-event-queries (and thus simple drug-event-findings) to a higher level (e.g., class effects, syndromes). Correct mapping of ontologies to the raw pharmacovigilance data will improve data quality and query construction as well as the information content of the results. Visualization of these outputs is under development for OpenVigil 2, AERS*Mine* and CzeekV. This improves the comprehension of the results and allows the application of other signal mining techniques, e.g., self-organizing maps [5].

The second important milestone, which is addressed by the software solution presented here, is the delivery of easy-to-use interfaces and fully automated calculations for common analysis scenarios, either scientific or clinical.

Here, we firstly present the OpenVigil FDA software and several “real-life” applications. OpenVigil FDA includes several pre-defined analysis scenarios for common clinical problems. These algorithms are described in detail below; results from various clinical situations are displayed and compared to other findings from the literature.

## Material and Methods

OpenVigil FDA consists of a single program file written in the PHP (PHP Hypertext Preprocessor) programming language. OpenVigil FDA

1. delivers a user interface for general data extraction, counting or analysis of reports as well as several specialized interfaces for clinically relevant scenarios, such as drug-drug interactions (DDI) and the comparison of the safety profiles (i.e., the entirety of all AE reported with a drug) of two or more drugs,
2. processes the request and builds one or several queries for the online API at https://api.fda.gov/drug/event.json to retrieve the reports or counts needed for further analysis from the FDA openFDA database and computes so-called contingency tables or disproportionality analyses and
3. presents the data in tables in various output formats like human-readable HTML, an Excel-like spreadsheet with a horizontal table (comma-separated values, CSV) or various other forms (JSON, XML) to the user.

A spreadsheet program can use the output to produce the diagrams shown in the results section.

### PHP program for web GUI, query processing logic and file exporters

The PHP script provides the user interface via the web, e.g., our installation at http://openvigil.pharmacology.uni-kiel.de/openvigilfda.php. After user interaction, a query to openFDA is constructed by using the API parameters “search”, “count” and, for pagination, “limit” and “skip”. The constructed query of each data retrieval can be manually inspected in the resulting HTML page, e.g., “?search = (patient.drug.openfda.substance_name:diazepam+AND+haloperidol)+AND+patient.reaction.reactionmeddrapt.exact:pain”.

Some search modes require repeated retrieval of values. This is hardcoded for the analyses scenarios shown below. Background-correction via indication or other masks [6, 7] is available.

OpenVigil FDA offers access to individual records or it counts records for a selection of safety reports, e.g., based on the openFDA data fields for pharmaceutical product (“medicinalproduct”), active ingredient/drug (“substance_name”, referred to as “drugname” in the OpenVigil software packages), generic name (“generic_name”) using U.S. adopted names (USAN), adverse event (“reactionmeddrapt”) coded using preferred terms (PT) of the Medical Dictionary for Regulatory Activities (MedDRA), indication (“drugindication”) coded using MedDRA and others. Users can pass their own query-string to openFDA and use OpenVigil FDA to display and analyze the result. Individual reports can also be accessed by their safety report identifier (“safetyreportid”).

The data retrieval logic of OpenVigil FDA was successfully validated against the official FDA demos [8].

### Algorithms for disproportionality analysis

The aim of pharmacovigilance is to find disproportionality in the spontaneously gathered reports. Disproportionality refers to the “overrepresentation” and “underrepresentation” of a particular group in the data, e.g., reports concerning a certain drug and event are more frequently filed than all other combinations of drugs and events.

#### 2x2 Contingency table of drug vs event

**Fig 1:**
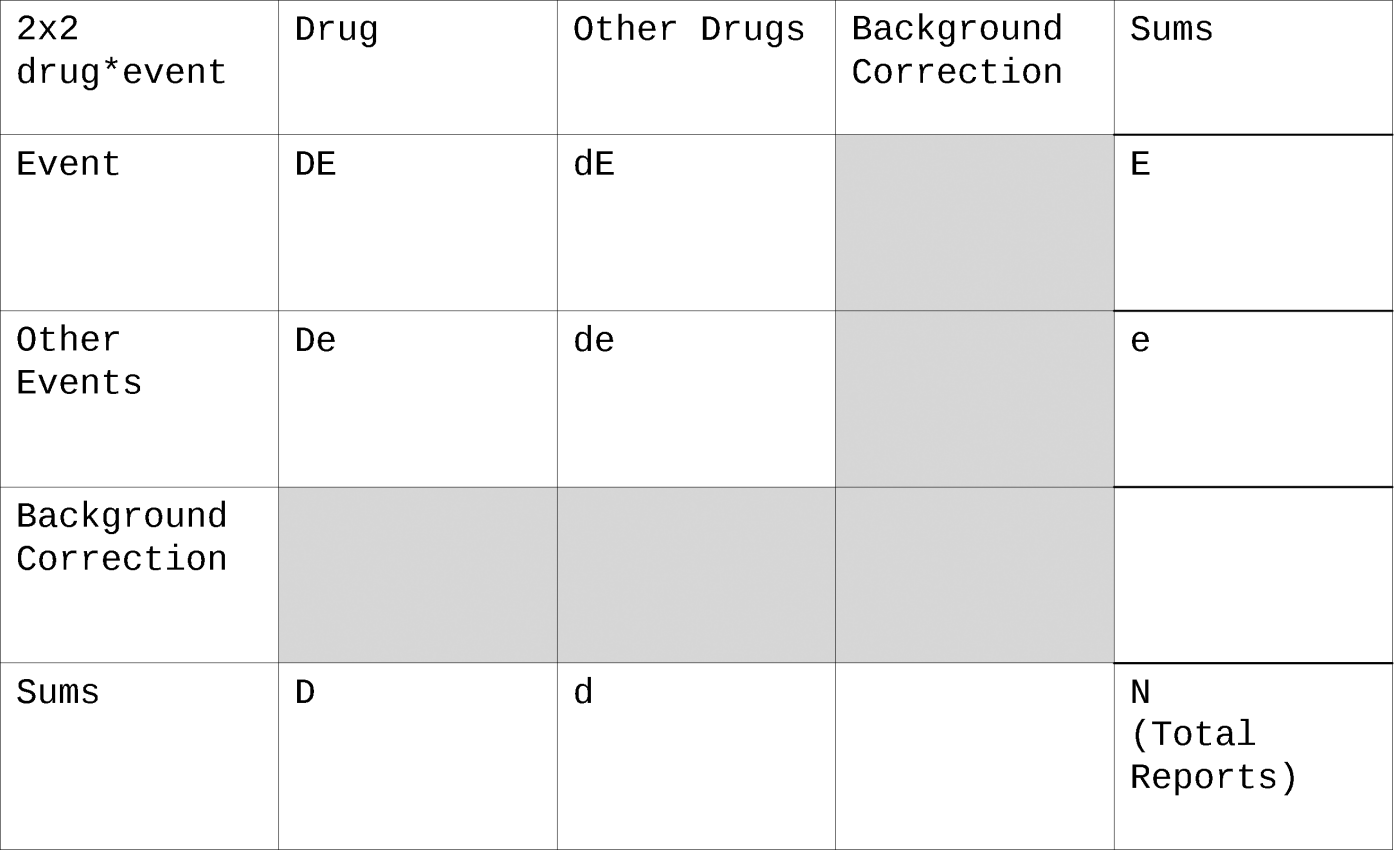
2x2 contingency table. The letters “D”, “d”, “E” and “e” refer to the datasets involved: Capital D: drug was used, lowercase d: this drug was not used but other drugs, Capital E: event occurred, lowercase e: this event did not occur but other events. For more complex contingency tables, indices can be used to refer to a certain drug or event by name or numbering. Intersections of the datasets can be made by combining letters, e.g., DE is the subpopulation where the drug was used and the event occurred. More details see text and Table 3.

The basis for all further calculations, i.e., the disproportionality analysis (DPA), is a 2x2 contingency table of the two categorical variables drug usage (D) and event occurrence (E) (Fig 1):

A capital D denotes exposition to a certain drug, a lowercase d no drug exposition. A capital E denotes occurrence of an adverse event, a lowercase e no occurrence of the event. Intersections of these datasets can be made by combining letters, e.g., DE is the subpopulation where the drug was used and the event occurred (Table 3). Letters can refer to the set or the number of records in this set. This naming convention for the cells of the table is preferred over using A-D or n_xy_ since some statistical measurements like the proportional reporting ratio (PRR) are vulnerable to transformations of the table (does the cell B stand for De or dE?). Furthermore, this naming scheme can easily be expanded to accommodate more than one drug or event by using letters with indices. E.g., D_1_d_2_E_1_E_2_ refers to the intersection of D_1_ (drug 1 used), d_2_ (drug 2 not used), E_1_ (event 1 occurred) and E_2_ (event 2 occurred), in other words: in this population drug 1 was used but not drug 2 and both the events 1 and 2 occurred.

**Table 3:** Naming of cells in the 2x2 contingency table.

Cell	Reference to a set of reports or the number of reports where…
D	the drug was used
E	the event occurred
DE	both the drug was used and the event occurred
de	neither the drug was used nor the event occurred
dE	the drug was not used but the event occurred
De	the drug was used but the event did not occur

#### Measurements of disproportionality

**Fig 2:**
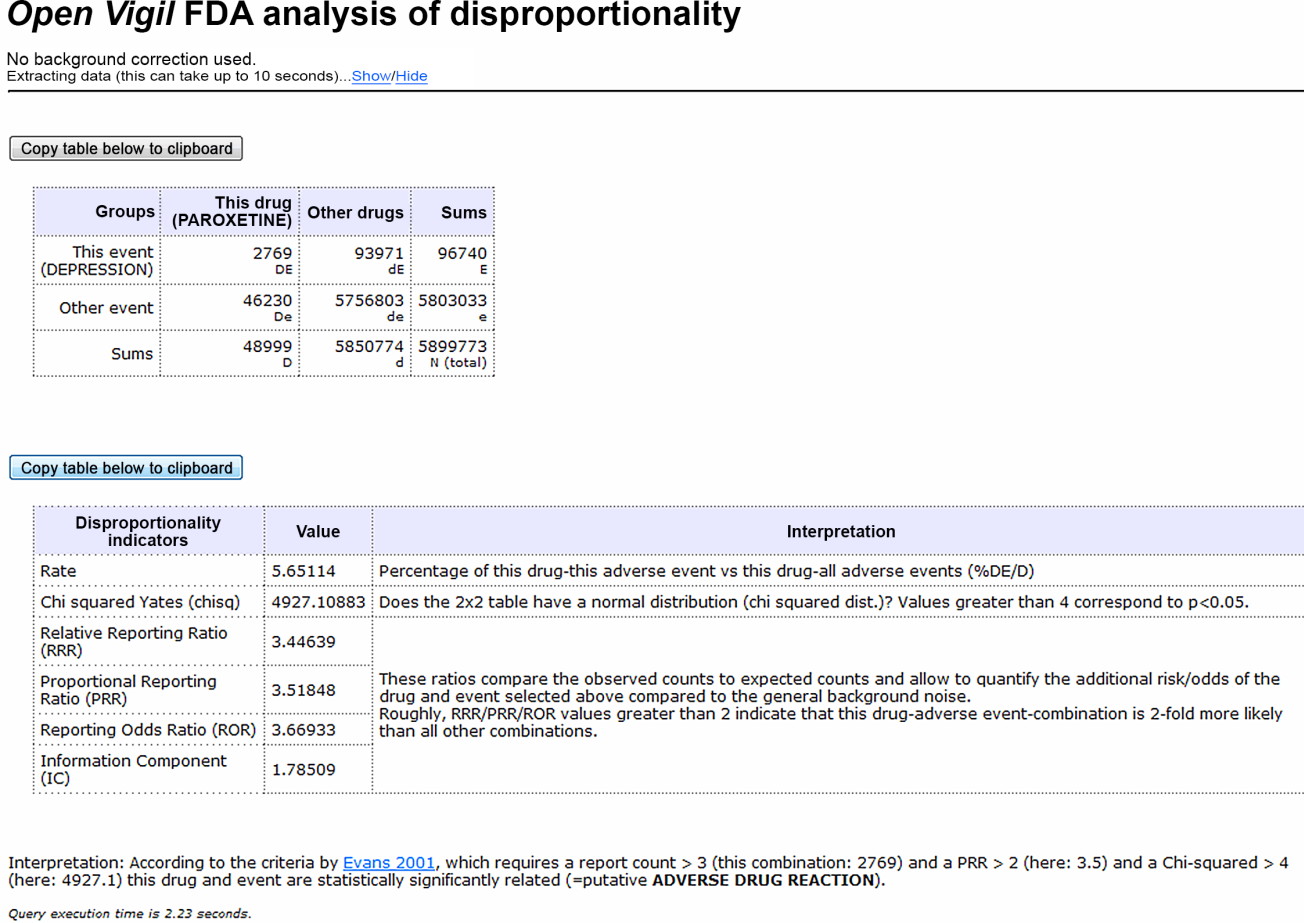
OpenVigil FDA: 2x2 disproportionality analysis (DPA) output. The letters “D”, “d”, “E” and “e” refer to the datasets involved. Chisq ($\chi_{Yates}^{2}$), RRR, PRR, ROR are measurements of disproportionality (calculations see text). Cf. the OpenVigil tutorials and caveat document how to interpret this interesting signal suggesting that an antidepressant causes depression. The signal is probably due to a mixture of wrong reporting (e.g., the field for event was used instead of the field for indication in the reporting form), drug failure (events "depression" + "drug ineffective") and a certain vulnerable subpopulation. Access date 2016-03-15.

Fig 2 shows an example and the necessary openFDA queries for a simple one drug (“paroxetine”) and one adverse event (“depression”) DPA design.

If the given drug and adverse event (AE) are disproportionally high represented in the database, the AE might be a true adverse drug reaction (ADR). This has to be confirmed through further pharmacologic research. It is, however, not sufficient to calculate the percentage (“rate”) of reports where both the drug was used and the event occurred (DE) of all reports where the drug was used (D):

$$Rate=\frac{DE}{D}$$ (1)

This number must be set in relation to all other rates in the database. Disproportionality is calculated using various formulas of statistics for clinical trials or cohort studies [2]. Currently, the following measurements of disproportionality are implemented:

The Odds Ratio (OR) is adapted as Reporting Odds Ratio (ROR) and calculated using

$$ROR=\frac{DE\times de}{dE\times De}$$ (2)

This addition of “reporting” to the name hints at the open-world problem of this data which is discussed later. Briefly, absolute rates or ratios as known from clinical studies can never be calculated correctly since the correct denominator (i.e., knowledge of all the population, including non-users of the drug) is missing (cf. the statistical considerations section below).

The relative risk (syn. risk ratio) is adapted as Proportional Reporting Ratio (PRR), and calculated using

$$PRR=\frac{DE\times d}{dE\times D}$$ (3)

The Relative Reporting Ratio (RRR) is calculated using

$$RRR=\frac{DE\times N}{E\times D}$$ (4)

When used on real-world data, ROR, PRR and RRR deliver very similar results (Fig 2). A value of 1 suggests that there is neither a disproportional association nor dissociation of drug and event. E.g., DE = 1, D = 10, E = 10, de = 81 describes a population where the event occurred in 10% of the drug users (DE/D) but also in 10% of the users of other drugs (dE = E—DE = 9; d = dE + de = 90, dE/d = 10%) (S1 Fig). Thus, all measurements of disproportionality are 1 in this case. Changing these numbers to DE = 2 and de = 72 models a population where 20% of drug users had an event while only 10% of users of others drugs experienced this event. RRR = 1.8, PRR = 2 and ROR = 2.25 in this situation (S2 Fig). PRR is routinely interpreted as a factor of frequency of an event occurring in the population of interest, i.e., users of this drug. PRR = 2 describes that the event is two times more frequent in population of users of the drug than in the background population. Since all three measurements of disproportionality are similar, RRR is used for all further calculations and the authors suggest that the RRR has the same explanatory power as the other two measurements.

The Chi-squared value (χ^2^ with Yates' correction) is also calculated to prove a non-normal distribution in the 2x2 table [9, 10]:

$$\chi_{Yates}^{2}=\frac{N\times {\left(\left|\left(DE\times de)-(dE\times De\right)\right|-\frac{N}{2}\right)}^{2}}{\left(E\times e\times D\times d\right)}$$ (5)

Evans suggested PRR > 2, $\chi_{Yates}^{2}$ > 4 (= p < 0.05) and DE > 3 as minimal criteria for a signal of disproportionality [9].

The formulas used in OpenVigil FDA were successfully validated against R/epiR 0.9–69 and web-based contingency table calculators [11–14].

The FDA, the WHO, OpenVigil and various other institutions provide caveat documents and/or disclaimers concerning the interpretation of these analyses due to the quality and bias of the data and method of data acquisition.

There is a significant increase in data retrieval and computing work for more complex DPA than the simple 2x2 design (calculation time: <4 seconds) described above. Therefore, OpenVigil FDA currently offers only two of these modes of analyses whose calculation time can take up to two to three minutes. These analyses are a complex analysis of one adverse event and several drugs (used for the first algorithm presented) and a complex analysis of two drugs on all mutual adverse events (used for algorithms 2 and 3).

### Clinical scenarios

The following algorithms based on DPA can be used to generate these hypotheses:

1. Which drug of a medication list should be discontinued first based on the relative reporting ratio (RRR) of this drug and the observed adverse event?
2. Which drug from a certain drug class should be favored for an individual patient due to its adverse event safety profile?
3. Can we detect any drug-drug-interactions (synergism/antagonism) by comparing the adverse event safety profiles of each drug and their combination?

To answer these questions, the values for DE, D, E and N are extracted and rates (cf. Eq 1) and RRRs (cf. Eq 4) are calculated.

### Algorithm 1: Which drug to discontinue first?

OpenVigil FDA extracts N and E for a given adverse event. For every drug listed, DE and D are extracted. These values are sufficient to calculate rates and RRR. The drug with the highest rate resp. RRR should be considered for being stopped first.

### Algorithm 2: Which drug out of two to prefer for an individual patient?

OpenVigil FDA extracts N and D_1_ and D_2_ and D_1_D_2_ with the index referring to the drug in question. After that, D_1_D_2_ is subtracted from D_1_ and D_2_, so only cases with either drug but not their combination are considered. Furthermore, all D_1_E_x_ and D_2_E_x_ with x being an adverse event reported for either of both drugs are extracted. Rates and RRRs for each adverse event and each drug are calculated. A large difference between the rates or the RRR of drug 1 and of drug 2 is indicative for adverse events that are predominantly associated with only one drug but not the other. Absolute differences might be misleading: A difference of 1 can be of significance (e.g., RRR_D_1_E_x_ = 1, RRR_D_2_E_x_ = 2) or probably by chance (e.g., RRR_D_1_E_x_ = 100, RRR_D_2_E_x_ = 101). Therefore, the percentage difference was used and calculated as

$$RRR\_perc\_diff=\frac{RRR\_D_{1}E_{x}-RRR\_D_{2}E_{x}}{mean(RRR\_D_{1}E_{x}-RRR\_D_{2}E_{x})}\times 100\%$$ (6)

By calculating these percentage differences of the RRRs compared to the mean of the RRRs we have chosen the most extreme outliers, e.g., less than -75% or greater than +75% as cut-offs, and arrange these data in Microsoft Excel 2003 for visualization (S1 Dataset). The cut-off values were chosen arbitrarily to catch the top 12–15 events with the largest differences between both drugs. There is currently no consensus which cut-offs are clinically or otherwise significant.

### Algorithm 3: Are there drug-drug interactions (DDI)?

OpenVigil FDA does the same extraction process as described above. This time, however, not the difference between the drugs, but rather between both drugs alone and both drugs in combination is of relevance:

$$Rate\_diff=(Rate\_D_{1}E_{x}+Rate\_D_{2}E_{x})-Rate\_D_{1}D_{2}E_{x}$$ (7)

If the observed rate of an event for the combination (D_1_D_2_E_x_) is either much smaller or much larger than the expected rate (sum of the rates for each drug used alone: D_1_E_x_ + D_2_E_x_), an interaction is likely. Similar to the algorithm above, there is no consensus on clinical cut-off values. Therefore, they were chosen arbitrarily to catch the top 16 events with deviating rates of occurrence.

## Results

All data extractions were done on 2015-10-26. At that time, the database contained 4,883,922 reports (S3 Fig). All reports where used as background for the calculations below. Only 88.08% of these reports contained at least one cleaned drugname. Of note, this does not imply that they have been completely cleaned (see below). The ongoing work of cleaning of drugnames will influence the results.

### Application 1: Risk for an adverse event from a medication list

**Fig 3:**
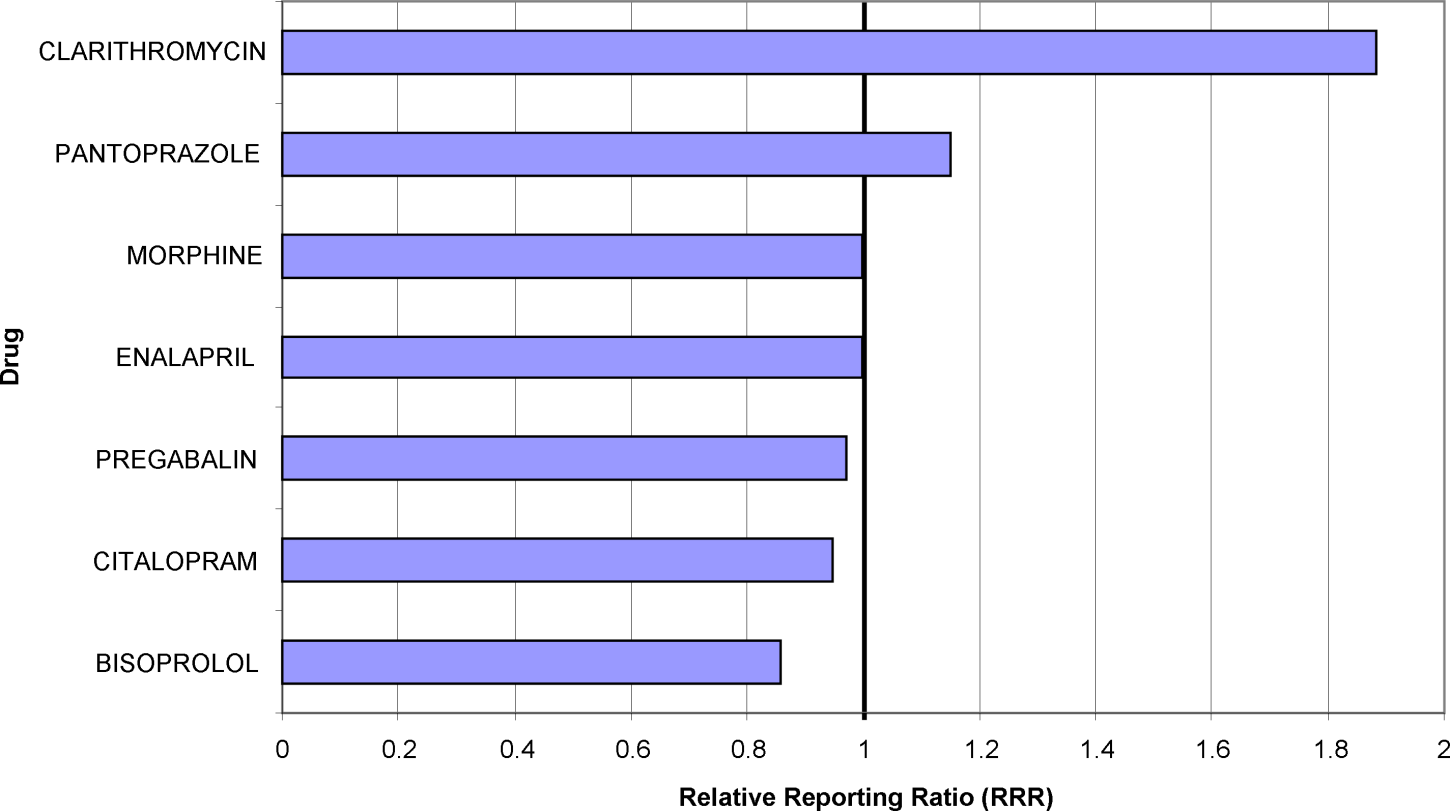
Relative Reporting Ratios (RRR) of the adverse event “rash” for a given list of drugs. A RRR value of 1 indicates the normal background noise, e.g., associations by chance. An increase of RRR indicates an overproportional association between the drug and the adverse event. RRR values much lower than 1 indicate a negative association, e.g., the usage of the drug protects the patient from the adverse event.

OpenVigil FDA provides an easy to use interface to check a newly occurring adverse event against a medication list. As an example, entering the adverse event “rash” and a typical medication list yields the data for the diagram shown in Fig 3. The Relative Reporting Ratio (RRR) is used to compare the strength of the association of one of these drugs with the event. The data imply that in this given case clarithromycin is the drug with the highest RRR and therefore could be discontinued first (and, of course, be substituted for with another antibiotic, if necessary).

### Application 2: Comparison between drugs–which drug to prefer for an individual patient?

A drug class usually consists of many drugs with similar or even identical clinical effects. Which drugs should be preferred, either generally or for a patient with individual comorbidities? We give three examples for comparing drugs, either similar for indication or for pharmacodynamic action. Assuming that drug 1 and drug 2 are equally effective for a certain indication, pharmacovigilance can be used to compare the AEs: The RRR is used to compare the strength of the association of either drug with all events found in the database. A marked difference of the RRR for drug 1 and an event and of the RRR for drug 2 and the same event implies a difference in the safety profiles (i.e., the entirety of all AEs/putative ADRs) of these drugs.

#### Imatinib versus Nilotinib

**Fig 4:**
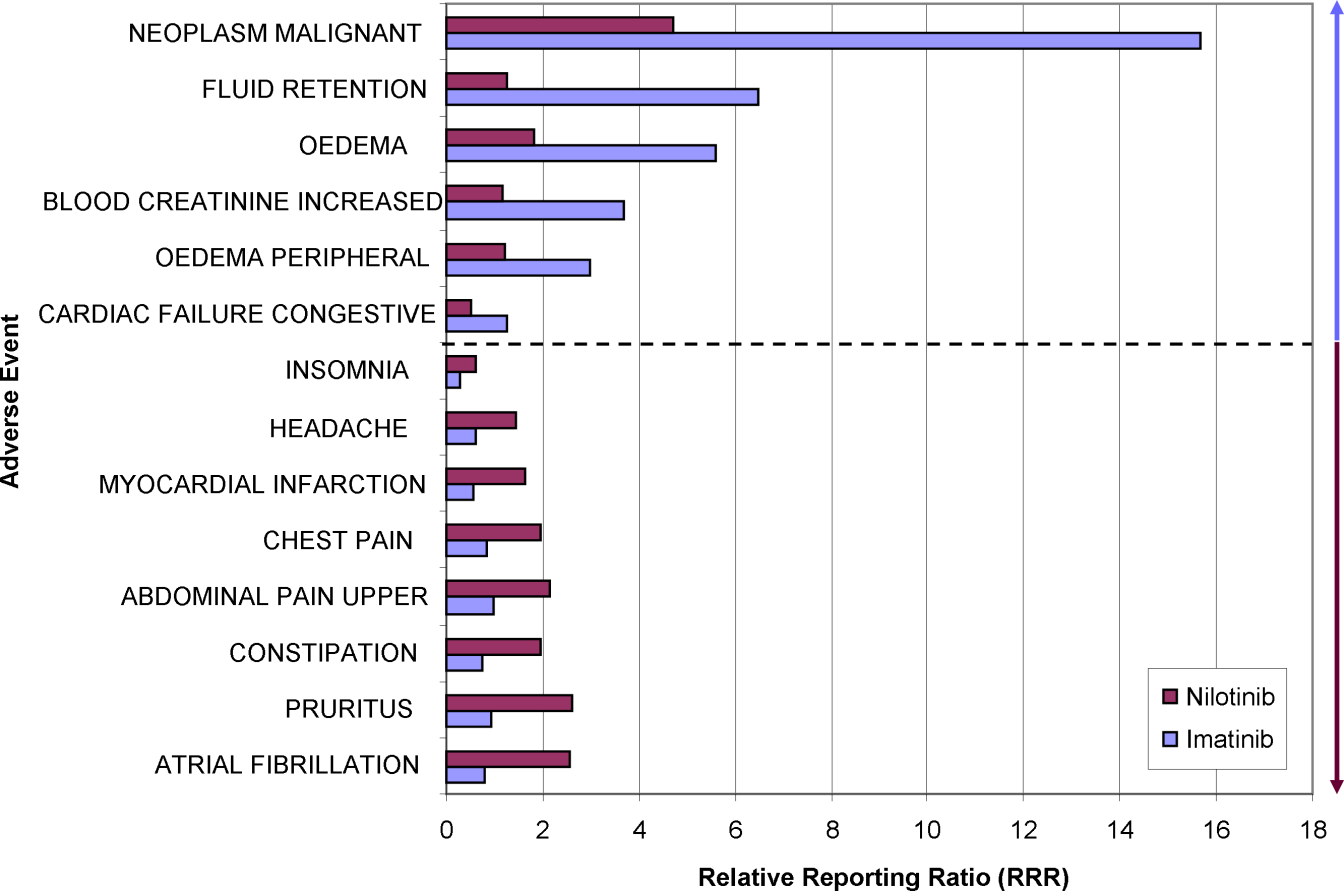
Comparative adverse event-profile of two tyrosine kinase inhibitors. The largest relative differences in the RRRs of either nilotinib or imatinib are visualized for selected adverse events. The blue arrow indicates adverse events which are stronger associated with imatinib than nilotinib, the ruby arrow vice versa. Both groups are separated by the dotted line.

Both imatinib and nilotinib inhibit BCR-ABL tyrosine kinase and are used for the treatment of chronic myelogenous leukemia. A comparison of their safety profile, i.e., percentage differences of the RRRs for imatinib and nilotinib for all listed events in the database, with cut-offs of less than -75% or more than 75% is shown in Fig 4. While “NEOPLASM MALIGNANT” is probably caused by confounding factors, i.e., the underlying disease, several other AE reveal a particular risk for one of the two tyrosine kinase inhibitors. Here, several types of oedema appear to be stronger associated with the use of imatinib than nilotinib. This is in accordance with a previous clinical trial [15]. On the other hand, cardiac problems are more often reported for users of nilotinib and much less for imatinib. The data of clinical trials did not allow the statistical calculation of differences in QT-time prolongation due to the low number of cases [15]. Pharmacovigilance data holds the promise to be especially useful for analyzing infrequent events.

In consequence, imatinib should not be prescribed for patients with renal disease whereas nilotinib should be avoided in patients with a history of cardiac disorders, e.g., long QT-syndrome or coronary heart disease.

#### Gabapentin versus Pregabalin

Gabapentin and pregabalin are primarily antiepileptic drugs which are predominatly used as analgetics in neuropathic pain and sometimes as mood stabilizers. To our best knowledge, there is no randomized controlled clinical trial that was explicitly designed to detect differences in the safety profile of gabapentin and pregabalin. There are two small trials comparing efficacy and safety after switching from gabapentin to pregabalin [16, 17]. Pharmacovigilance data contain more cases for either drug and are thus ideally suited for comparing the safety profiles.

**Fig 5:**
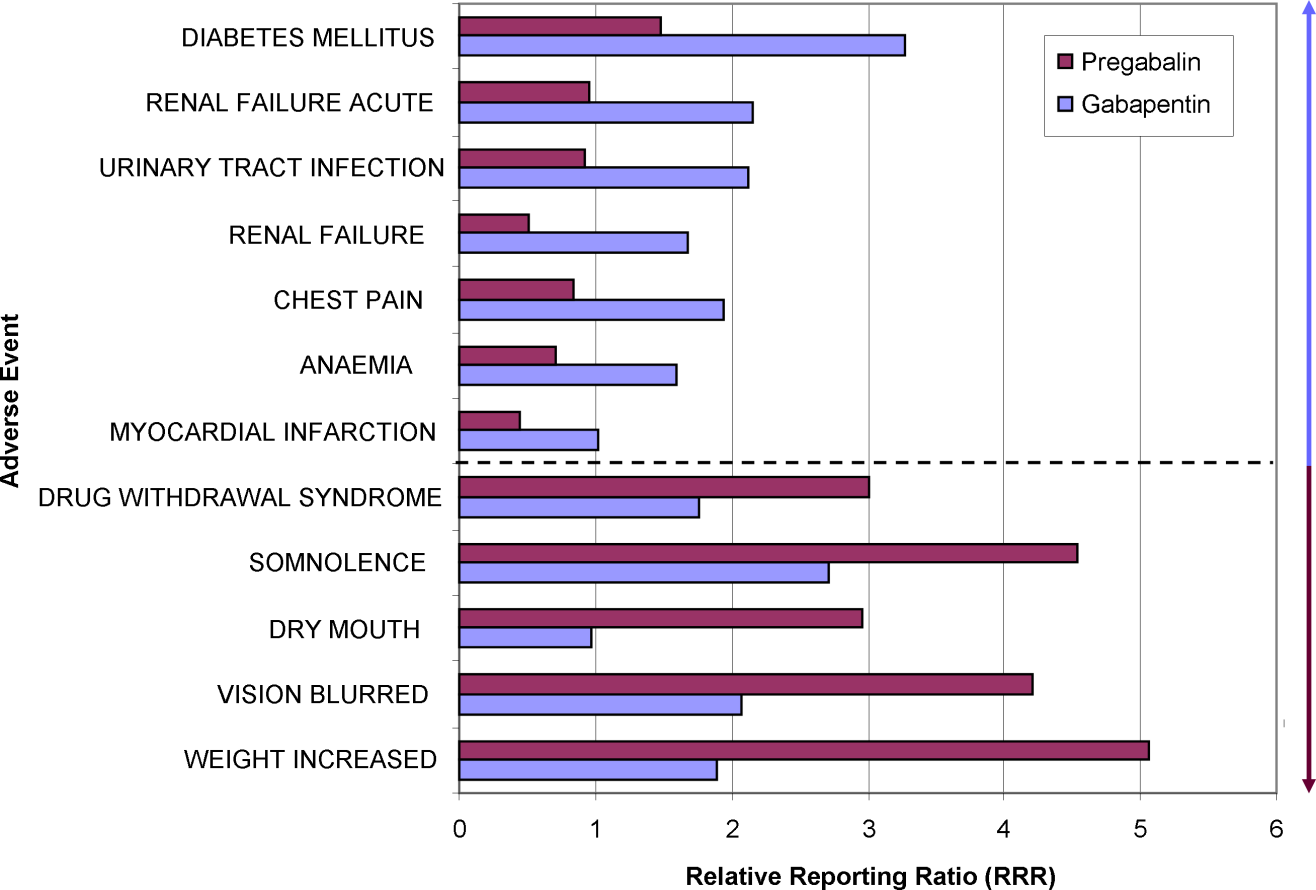
Comparative adverse event-profile of two drugs used for neuropathic pain, epileptic seizures and mood stabilization. The largest relative differences in the RRRs of either gabapentin or pregabalin are visualized for selected adverse events. The blue arrow indicates adverse events which are stronger associated with gabapentin than pregabalin, the ruby arrow vice versa. Both groups are separated by the dotted line.

Such a comparison of the safety profile (percentage differences of risks for an AE) of gabapentin and pregabalin with cut-offs of less than -50% or more than 75% is shown in Fig 5.

It is evident that weight increase is linked with the use of pregabalin. Depending on the comorbidities of the patient, gabapentin should be preferred for obese patients. The most frequent indication for all the reported cases with pregabalin and weight increase is fibromyalgia. This could be a confounding factor, since fibromyalgia itself is associated with obese patients and prospective weight increase. However, background correction using either “fibromyalgia” or “depression” as indication does not substantially alter this signal.

Interestingly, we had previously identified a complex of anxiety, depression, suicidal ideation, suicide attempt, completed suicide and even the AE “GUN SHOT WOUND” associated with use of gabapentin [18]. However, since each single preferred term is not included in the range of this selection (cut-off for all openFDA results to 100 records), these signals were not detected. Furthermore, depression as confounding factor needs to be evaluated. Gabapentin and pregabalin are equally frequently used in patients with the indication “DEPRESSION” in the openFDA data. Re-extracting the safety profiles by using this background correction does strengthen the signal for “SUICIDE ATTEMPT” for gabapentin in comparison to pregabalin from no findings in the uncorrected data to a 97% difference between RRRs in the background-corrected data.

Compared with our previous results, the association of “OEDEMA PERIPHERAL” and “FEELING ABNORMAL” with pregabalin was not detected here. “OEDEMA PERIPHERAL” is around 40% less common with gabapentin (S1 Dataset). This is in line with findings from clinical trials [16] and from an analysis done using OpenVigil 2 [18] but the signal was not considered due to the cut-off values employed for this analysis (< -50%).

#### Ondansetron versus Metoclopramide

**Fig 6:**
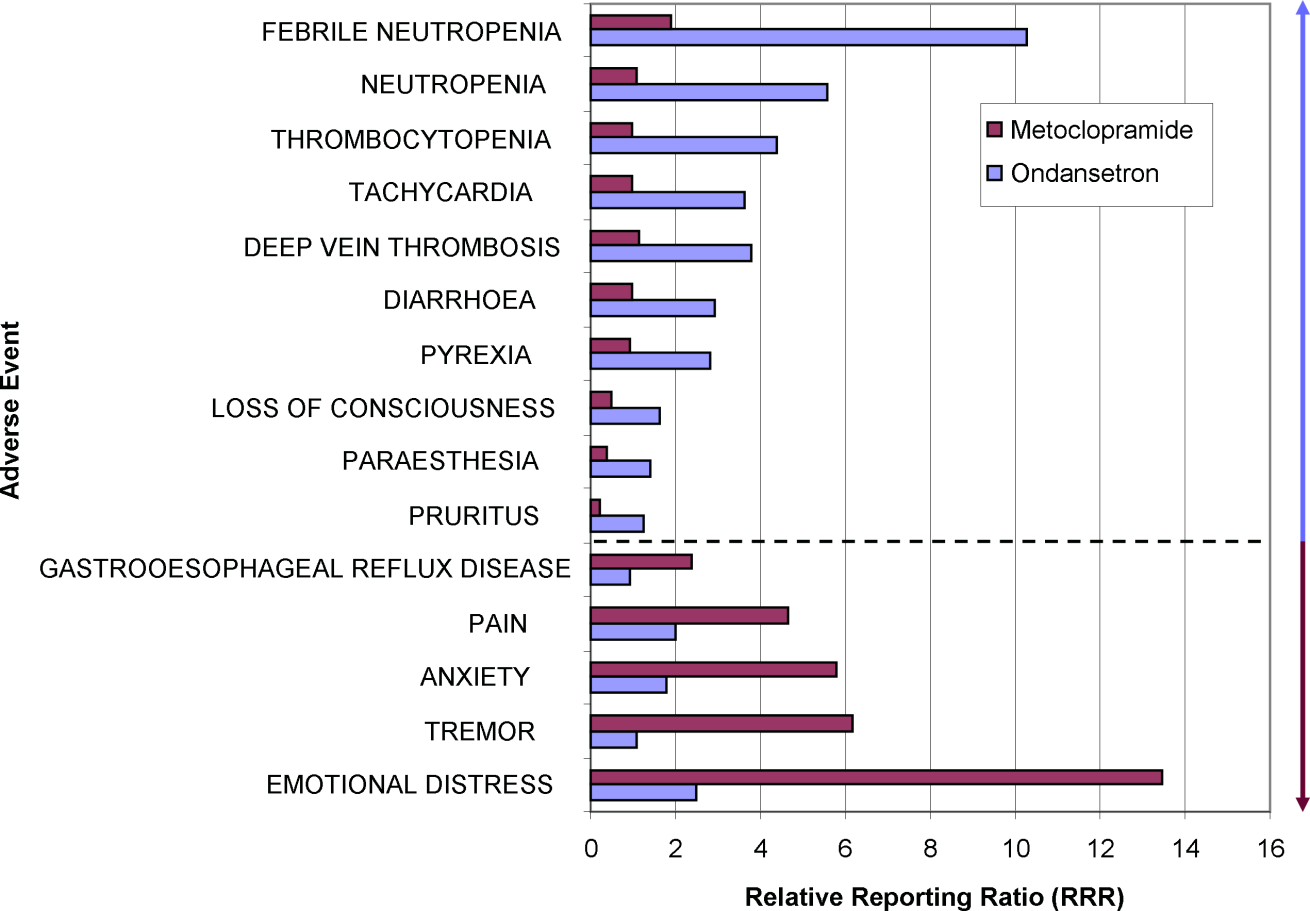
Comparative adverse event-profile of two antiemetic drugs. The largest relative differences in the RRRs of either ondansetron or metoclopramide are visualized for selected adverse events. The blue arrow indicates adverse events which are stronger associated with ondansetron than metoclopramide, the ruby arrow vice versa. Both groups are separated by the dotted line.

Nausea and emesis are common problems after surgery or during drug therapy, in particular during chemotherapy or treatment with opioids. Assuming that these types of nausea can be equally well treated by the 5-HT_3_-antagonist ondansetron and the D_2_-antagonist metoclopramide which act on slighty different pathways of emesis, a comparison of the drug safety profiles (percentage differences of risks for an AE) provides further suggestions which individual vulnerabilities of the patient should be considered (Fig 6): Metoclopramide is strongly linked to neuropsychiatric adverse events most likely explained by its central antidopaminergic action. The relatively stronger signals for (febrile) neutropenia and thrombocytopenia for ondansetron might be due to the confounding chemotherapy treatment which in turn is usually accompanied by prophylactic or pro rea nata prescription of ondansetron.

In contrast to previous findings from randomized controlled trials that ondansetron slows intestinal transport and reduces the symptoms of irritable bowel syndrome, ondansetron appears to be associated with diarrhea in these data. We attribute this discrepancy to the underlying disease of gastrointestinal disorder or malignoma with subsequent chemotherapy that will affect the colon. In fact, ondansetron is frequently used in reports containing the indications of different types of cancer or “chemotherapy”. This explanation is in accordance with a study in patients receiving cisplatin and experiencing less adverse events, especially fewer loose stools, in the ondansetron group [19]. Since not every report contains data on indications, a background correction is not easily possible in this case.

Summarizing, metoclopramide should be avoided in patients with stress or neuropsychiatric disorders. This is in accordance with the results of a trial comparing ondansetron and metoclopramide for hyperemesis gravidarum which showed that the ondansetron group has fewer adverse events and that metoclopramide is associated with central adverse events like dizziness and drowsiness and with palpitations [20].

### Application 3: Drug-drug Interactions (DDI)

Drug regulating authorities require the manufacturer to provide in-depth pharmacokinetic and–dynamic (PK/PD) data for new drugs. Drugs which have been brought to the market before this requirement was in force are in general sufficiently characterized, too. These PK/PD data are used to generate hypotheses for DDIs. However, clinical experience has shown that a new DDI can turn up unexpectedly. Currently, there are approximately 2,000 substances available as drugs in Germany. Mathematically, approximately two million drug-drug interactions are possible. At the same time, a large German drug information portal lists just about 1,500 known DDIs. Some of these DDI monographs are on the level of drug classes, not of individual substances. False negative and false positive warnings frequently occur. Therefore it is imperative to establish other methods to detect DDIs. The measurements of disproportionality for drug 1 and an event (D_1_E_x_) and the measurements for drug 2 and the same event (D_2_E_x_) are combined to extrapolate expected measurements of disproportionality for the combination of both drugs and this event. A difference between this extrapolation and the observed numbers (D_1_D_2_E_x_) in the database indicates some kind of interaction between these drugs with respect to this event.

**Fig 7:**
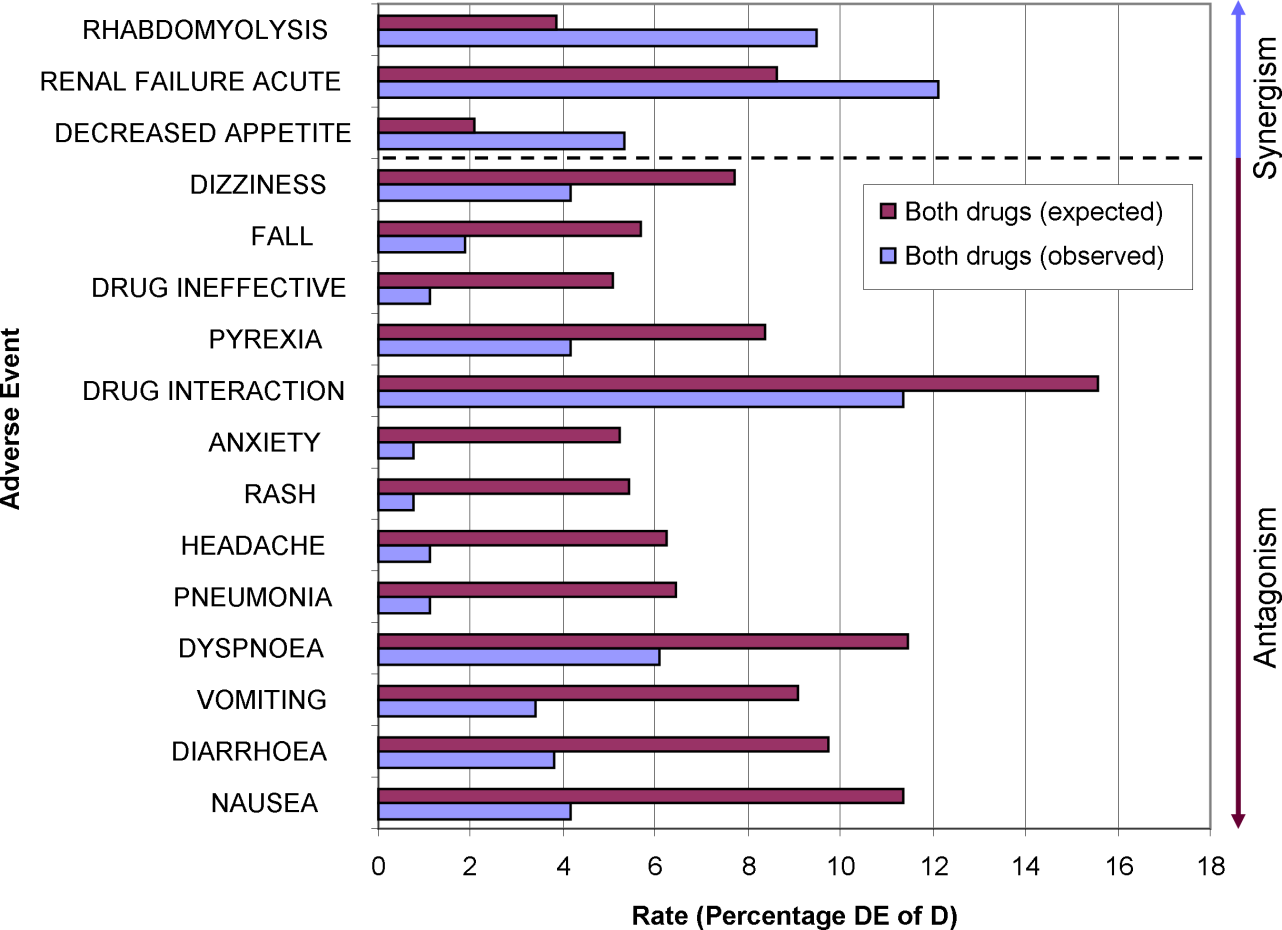
Comparison of expected-observed rates for two drugs and their combination. An observed rate that is greater than the expected rate suggests a synergistic interaction. Vice versa, an observed rate lower than expected suggests an antagonistic interaction.

As an example, clarithromycin and bisoprolol are two drugs commonly co-prescribed in our hospital. To our best knowledge, there is no randomized controlled clinical trial that was explicitly designed to detect unknown adverse events of this combinations. Instead, our current knowledge is based on case reports and pharmacological hypotheses. Besides the known and apparent DDI, are there any interesting unknown adverse events that are more or less common than expected, i.e., new DDIs? Differences of the additive rate for an adverse event for both drugs used alone (D_1_E_x_ + D_2_E_x_) and of the rate for their combined usage (D_1_D_2_E_x_) were selected if less than 3% or greater than 3.5%. These expected and observed rates of this list were visualized in Fig 7. These data clearly show that physicians and pharmacists are aware of the high interaction potential of the strong cytochrome p450 isoenzyme 3A4 inhibitor clarithromcyin–there are fewer reports for “drug interaction” than expected. Another explanation might be that this textbook-interaction is actually less severe than anticipated. The Swiss drug interaction checker mediQ has recently downgraded the severity of this drug interaction. Most adverse events in Fig 5 are less common when using the combination of the drugs. E.g., these data could be used to speculate whether the combination is effective *against* rash.

Adverse events occurring more frequent than expected are rhabdomyolysis, renal failure and decreased appetite. We attribute this to the confounding factor of a more aggravated illness in patients who received both drugs. The rhabdomyolysis might be confounded by concurrent statin usage since neither clarithromycin nor bisoprolol are–based on our current pharmacological knowledge–likely to interact with muscles cells.

## Discussion

U.S. American pharmacovigilance data are available through the openFDA interface. We have developed the OpenVigil FDA software to easily access this interface, extract data and analyze it. OpenVigil FDA has analysis modes intended to be used by physicians and hospital pharmacists to assist with common “real-life” clinical problems at bedside. The results of the different possible applications are discussed below.

### Application 1: Risk for an adverse event (AE) from a medication list

As show in Fig 3, the extracted data allow to rank drugs according to their RRR, i.e., according to their association with a defined AE. This ranking supports the decision which drug to discontinue first if a given AE is detected during and attributed to drug therapy. Traditional approaches for decision making were “eminence-based” medicine (i.e., ask experts) and to consult summary of product characteristics (SPC). The Side Effect Resource “SIDER”, for example, provides a quick overview of SPCs and official numbers from clinical trials or recommendations from national or European drug regulating authorities [21]. However, SPCs do very often contain misleading medical information, e.g., phony occurrence rates, either too low or too high, intended to protect the pharmaceutical company from legal proceedings. Thus we have previously commented warnings in the SPC of orlistat which do not base on a statistical nor pharmacological rationale [22]. Results of clinical trials that are performed or published under the contribution or co-authorship of pharmaceutical companies are biased, too. The need of data resources that do not depend on any numbers filtered by pharmaceutical companies, insurance companies or hospital networks is obvious.

Pharmacovigilance data holds the promise of being such a resource for numbers which, in conjunction with prescription data, might deliver better estimations of risks. However, various issues and biases have to be considered (see below).

The authors have successfully identified putative causative agents in several cases at our hospitals with increased hepatic enzymes (i.e., pantoprazole as causative agent in 2 cases) or thrombocytopenia (i.e., levetiracetam as causative agent) out of a medication list (S4 and S5 Figs). In other cases, cefpodoxime was identified as causing thrombocytopenia and metamizol (USAN: dipyrone) as causing increased hepatic enzymes. Importantly, discontinuation improved the condition. A comparison with the ADR frequency data from the SPCs and SIDER revealed a high correlation for all methods. Pharmacovigilance offers finer graded differences between the drugs and is easier and much faster to access using OpenVigil FDA for this kind of question than the other sources which need manual browsing of the data [23].

Summarizing, OpenVigil FDA can optimize drug therapy safety if a new AE has occurred. Clinical trials to estimate the benefit of this technique are worth to be considered.

Of note, this and the other analyses do not take the dosages into account. Drugs given in extremely low or high dosages are likely not to interfere or have a more prominent impact, respectively.

### Application 2: Comparison between drugs–which drug to prefer for an individual patient?

By comparing the difference in the RRRs for drug 1 and drug 2 for all listed events in the database, we have contrasted the safety profiles of these drugs (Figs 4–6). Individual events which are much more frequently seen with one drug but not the other indicate differences of the safety profiles.

Pharmacovigilance data can be considered as a huge pool of clinical trials. Assuming similar quality of reports, similar rate of under-reporting and similar drug usage within a population, it is tempting to use the data to compare two or several drugs within their drug or indication class (Figs 4–6). This kind of analysis is offered by commercial providers to hospital pharmacies and can be extended by multiplying the apparently higher risk of a drug with the costs to cope with these adverse events, giving the extra costs for certain “dangerous” drugs.

The authors do not endorse this practice but rather stress that the above mentioned assumptions in most cases do not sufficiently apply. Depending on the quality of the data, distortions are possible, and due to the limitations of openFDA not all data are extractable. E.g., for comparing drug safety profiles, OpenVigil FDA extracts all D_1_E_x_ and D_2_E_x_ with x being an adverse event reported for either of both drugs. For each set of results, there is currently a limit of 100 records. Therefore, openFDA does not yet allow deep data analysis. We are missing many potential signals of differences between safety profiles.

Of note, our results using openFDA for the comparison of gabapentin and pregabalin are different from those using OpenVigil 2 which uses a different data cleaning procedure and is able to extract all data without restrictions [18]. Several indications for a higher risk of suicide which is coded with various MedDRA preferred terms are not available in OpenVigil FDA due to the 100 records limit of openFDA.

### Application 3: Drug-drug Interactions (DDI)

Fig 7 presents deviations from an expected rate of occurrence for a certain adverse event if two drugs are given concomitantly by adding up the individual rates of either drug. Deviations of the observed rate from the expected rate indicate an interaction. There is currently no consensus which deviation could be deemed clinically significant. Other statistical procedures use different methodologies [24–26]. OpenVigil FDA allows extraction of all relevant numbers to apply these as well.

Due to the 100 records limit of openFDA, interesting interactions might not be found in the available results from this datasource.

### Data quality issues

The FDA reports that “roughly 86%” of all records have at least one cleaned drugname [27]. This figure is sometimes erroneously interpreted as “86% of all records are completely cleaned” [28] which is not true. 2x2 contingency table calculations require not only a numbers of reports where a certain drug was used but also numbers of reports where this drug was NOT used. Thus, reverting the 86% figure, openFDA contains about 14% of cases with unknown medications. Even worse, of the remaining 86% only a minor part contains cleaned drugnames for the entire medication list. The inclusion of unprocessed data to the dataset severely distorts the complementary numbers for any contingency calculations. In consequence, signals will change depending on the policy how to treat unmapped verbatim drugnames.

Furthermore, there are obvious mismappings present in these 86% “cleaned data” (S6 Fig) [29]. AdverseEvents.com reports their cleaning rate of the raw FDA AERS data to be 95–98%. We found mismapping in this supposedly clean data, e.g., “Atorvastatin (blinded)” or “Atorvastatin (ngx)(atorvastain) Unknown” in the supplemental material [28, 30]. An early experimental version of OpenVigil 2 managed to automatically clean about 66.6% of all reports in the FDA AERS data from 2004 to 2012Q2 using Drugbank v3.5 data and drugs@FDA. Most reports were discarded because of unknown drugnames (cleaning rate for all drugnames: 38.59%).

Correctly mapping the pharmaceutical product to a unique active compound is an ongoing problem that also affects the OpenVigil project. The U.S. data is a verbatim text-string that requires processing because of spelling-errors etc. Freely available resources like RxNorm, DrugBank.ca, drugs@FDA are available but none offers a 100% sensitivity and 100% specificity match.

openFDA provides the National Drug Code (NDC), Structured Product Labeling (SPL), Unique Ingredient Identifier (UNII) and RxNorm Concept Unique Identifier (RXCUI) to identify pharmaceutical products, strength and size. In Germany, routinely the Pharmazentralnummer (PZN, engl. central pharmaceutical number) is used for this purpose. These systems might overcome some of these problems mentioned above. OpenVigil FDA provides searches by UNII. However, different salts and enantiomers and other subtle differences are still an ongoing challenge to every nomenclature. Furthermore, these identifiers are usually not collected and reported at time of pharmacovigilance data sampling. openFDA re-attaches *all possibly matching* identifiers to the raw data during the drugname cleaning process (S7 Fig), thus creating mismappings of one report to noninvolved manufacturers and their pharmaceutical products.

### Data retrieval issues

Currently, the openFDA API restricts retrieval of reports to 5100 (?skip = 5000&limit = 100) and for counts to 100. The FDA stated high server load for these restriction in a blog [31]. While this situation is likely to improve, incomplete lists affect the above presented calculations. Any associations with n < 50 to 300 reports (estimation) are likely removed from the set of results. Therefore, some interesting, otherwise statistically significant associations might be missed by this access to the FDA AERS data. This holds particularly true for comparisons of drugs and for finding drug-drug interactions which might be hidden in the missing data.

Since December 2015, the FDA is providing a download of the current openFDA source and data for users who want to retrieve more query results than 5100 by using the user’s own servers with a custom configuration. Interested users have to solve technical challenges like accommodating the 100 GB of openFDA data in the user’s own database system.

### Confounding factors

Any signal might be due to a confounding factor. E.g., a disproportionality analysis of drug “dexamethasone” and event “thrombocytopenia” results in a statistically strong signal (PRR = 7.09). Looking at concomitantly used drugs and indications reveals bone marrow damaging drugs like cyclophosphamide, bortezomib, or cisplatin. AERS Spider, a closed-source AERS analysis system, routinely identifies such confounders [6]. Background correction can eliminate confounders and helps to focus on the “right” subpopulation, e.g., usage of dexamethasone without any chemotherapeutic drug. OpenVigil FDA implements basic background correction, called PRR-by-therapeutic-area (PRR-TA) by Grundmark et al [7].

The examples of the comparison of gabapentin versus pregabalin and ondansetron versus metoclopramide demonstrate that background correction is imperative to analyze subgroups like depressive patients or to exclude chemotherapy patients, respectively. For a medical meaningful analysis, the detection of confounders (e.g., criteria for a signal) and the resulting corrections should be defined beforehand.

### Statistical considerations

**Fig 8:**
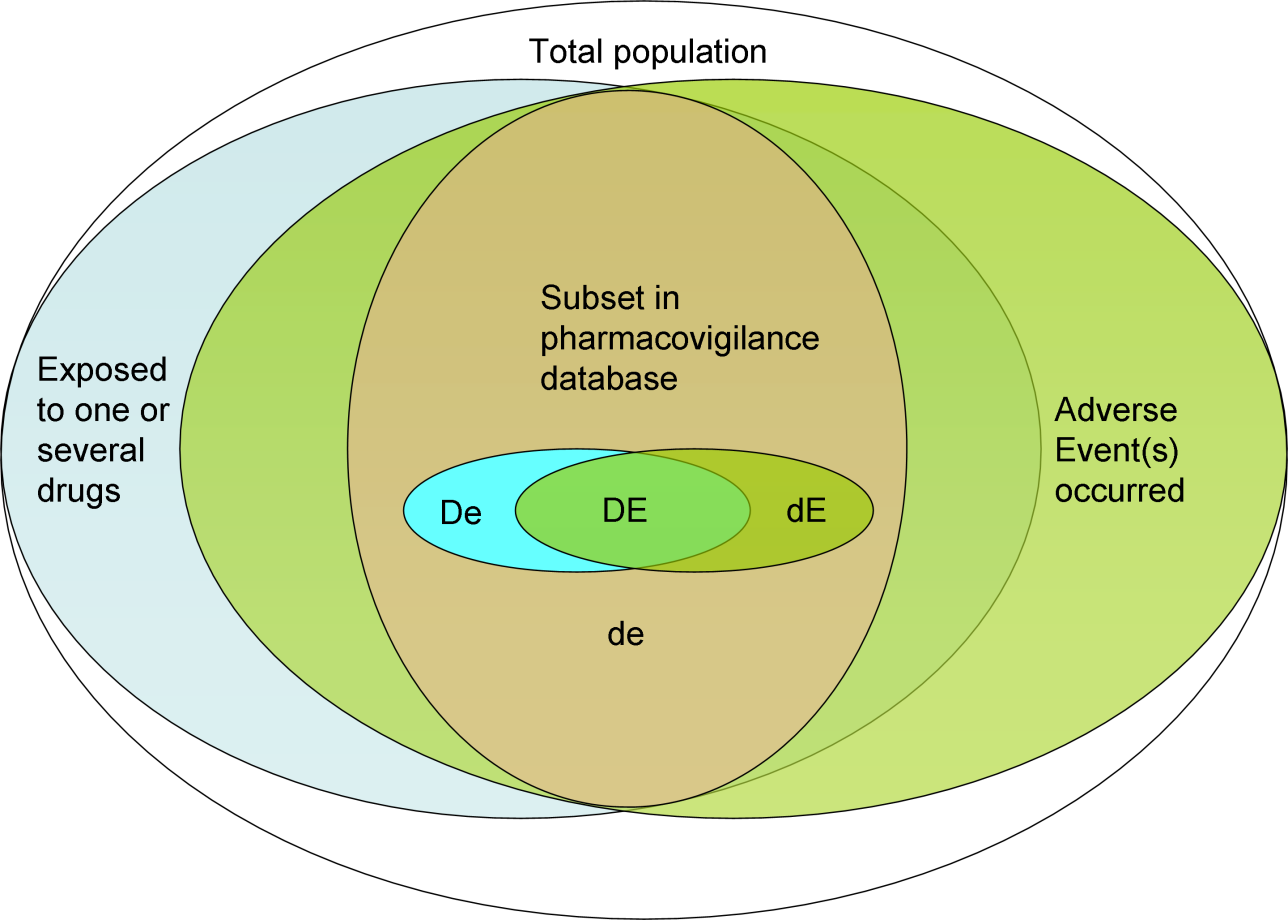
The open-world problem in pharmacovigilance. The numbers extractable from any adverse event database (DE, dE, De, de) come from a subset, i.e., all the reported cases. The rest of the population is unknown to the system.

Pharmacovigilance is an open-world problem, i.e., the recorded data are from a subset of the entire population (Fig 8). This implies that any findings are useful for hypothesis generation only. In principle, pharmacovigilance cannot prove anything. Strict statisticians will find the analyses proposed above to be deceptive. However, this approach is the best the current health care system can actually provide. Common clinical practice aims are minimizing potential damage to the patient wherever possible. Any signals found with OpenVigil FDA, although somewhat imperfect, can be used to stimulate further research on pharmacokinetics and -dynamics or for optimizing the medication of an individual patient when other sources of evidence or the product information are neither available nor sufficient.

## Conclusions

OpenVigil FDA offers an intuitive access for physicians and pharmacists to FDA AERS pharmacovigilance data. It provides custom user interfaces to address common clinical problems with better evidence than currently offered by existing resources like SPCs and SIDER.

OpenVigil FDA provides disproportionality analyses. OpenVigil FDA (i) identifies the drug most likely evoking a new adverse event, (ii) compares two drugs concerning their safety profile, (iii) checks arbitrary combinations of two drugs for unknown drug-drug interactions and (iv) enhances the relevance of results by identifying confounding factors and eliminating them using background correction.

Due to the limitations of the underlying openFDA data source, improvements to openFDA are necessary, or other analysis tools should be used for comparing the safety profiles of drugs or for finding drug-drug interactions. However, this does not invalidate any findings from OpenVigil FDA, since they must be considered as stimulating hypotheses, not as proofs.

OpenVigil FDA thus improves drug therapy safety: It is useful for clinical decision making once an adverse event has occurred, it allows selecting the best drug for patients with individual vulnerabilities and it helps to detect and avoid drug-drug-interactions.

## Supporting Information

S1 Dataset All raw data leading to the values used for Figs 3–7 as Open Document Spreadsheet (ODS). (ODS)

S1 Fig 2x2 contingency table and measurements of disproportionality for model case of no signal. (TIF)

S2 Fig 2x2 contingency table and measurements of disproportionality for a model case with signal. (TIF)

S3 Fig Overview of the software and database used with version numbers and dates. (TIF)

S4 Fig Which drug is most likely linked to an increase of serum gamma-glutamyl-transferase (gGT)? (accessed 2016-02-02).
(TIF)

S5 Fig Which drug is most likely linked to thrombocytopenia? (accessed 2016-01-07).
(TIF)

S6 Fig Mismapping of “ibuprofen” to a drug combination (diphenhydramine and ibuprofen). (TIF)

S7 Fig Fragment of the original JSON-data showing invalid multiple mappings. The drug “ibuprofen” is mapped to all possibly matching identifiers (here: manufacturer names, UNII, RXCUI, SPL_ID) resulting in non-involved products and manufacturers being associated with this report.
(TIF)

## Abbreviations

ADR: Adverse Drug Reaction
AE: Adverse Event
AERS: Adverse Event Reporting System
API: Application Programming Interface
CSV: Comma-Separated Values (data format
D: see also E; capital D denotes a set of reports where a certain drug was used; a lowercase d references a set without any usage of this drug; indices can be used to reference to a certain drug (e.g., D_1_ or D_orlistat_) and intersections of sets can be made by combining different D (or E [events]), e.g., D_orlistat_ D_doxazosine_ E_pneumonia_ describes a subgroup of patients on both “orlistat” and “doxazosine” with the adverse event “pneumonia”
DPA: Disproportionality Analysis
E: see also D; a capital E denotes a set of reports where an event occurred, a lowercase e references a set without this event; indices can be used and intersections can be made
FDA: Food and Drug Administration
HTTP: Hypertext transfer protocol (internet protocol)
JSON: JavaScript Object Notation (data format)
MedDRA: Medical Dictionary for Regulatory Activities
N: the total number of reports in the database or in a selection (e.g., background filter)
NDC: National Drug Code
OR: Odds Ratio
PHP: PHP Hypertext Preprocessor (programming language)
PRR: Proportional Reporting Ratio
PRR-TA: PRR by Therapeutic Area (background correction mask)
PT: Preferred Term (lowest terminology level of MedDRA)
PZN: Pharmazentralnummer (german), engl. central pharmaceutical number
R: a statistical programming language (successor of the statistical programming language S)
ROR: Reporting Odds Ratio
RRR: Relative Reporting Ratio
RR: Relative Risk or Risk Ratio
RXCUI: RxNorm Concept Unique Identifier
SIDER: Side Effect Resource (web-based database)
SPC: Summary of Product Characteristics
SPL: Structured Product Labeling
UNII: Unique Ingredient Identifier
URL: Uniform resource locator (“internet address”)
USAN: U.S. Adopted Name (for a drug)
