# Supplementary figures and images for: OpenVigil FDA – Inspection of U.S. American Adverse Drug Events Pharmacovigilance Data and Novel Clinical Applications
Source: PLoS One. 2016 Jun 21;11(6):e0157753. doi: 10.1371/journal.pone.0157753 (PMC4915658; doi:10.1371/journal.pone.0157753)

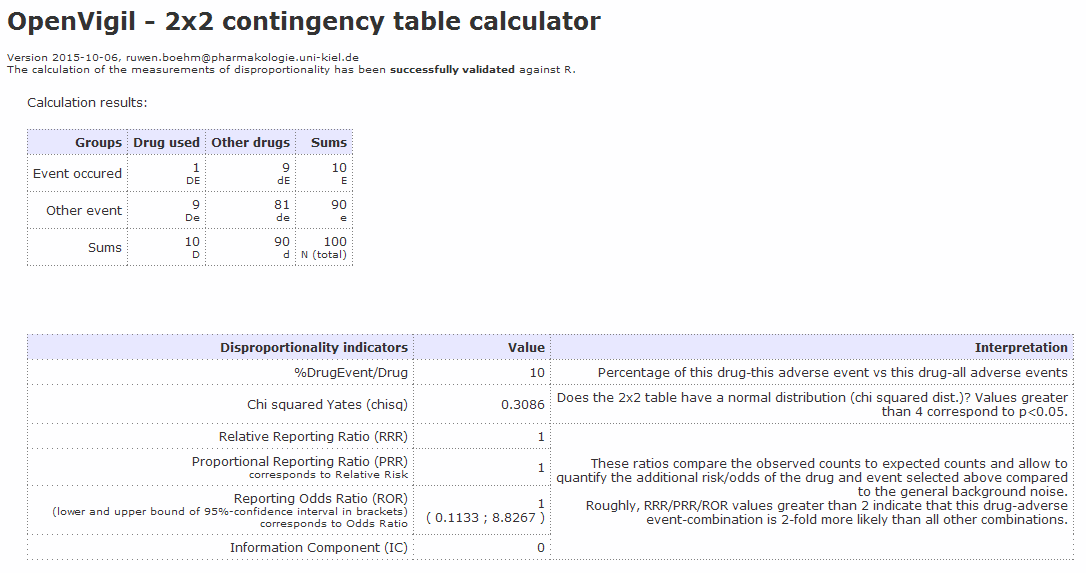

Supplement: S1 Fig — (TIF) [file pone.0157753.s002.tif]

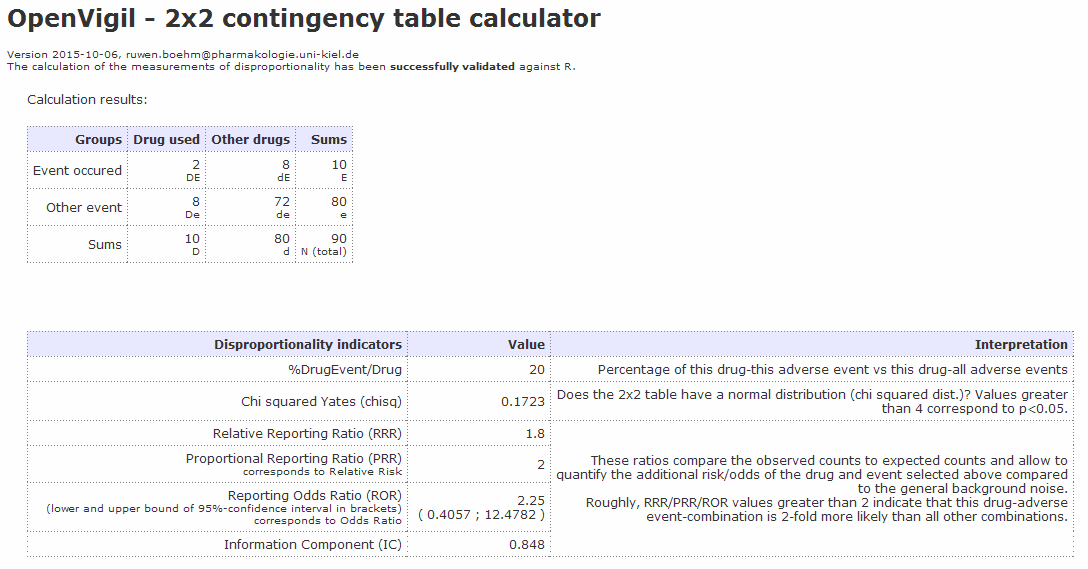

Supplement: S2 Fig — (TIF) [file pone.0157753.s003.tif]

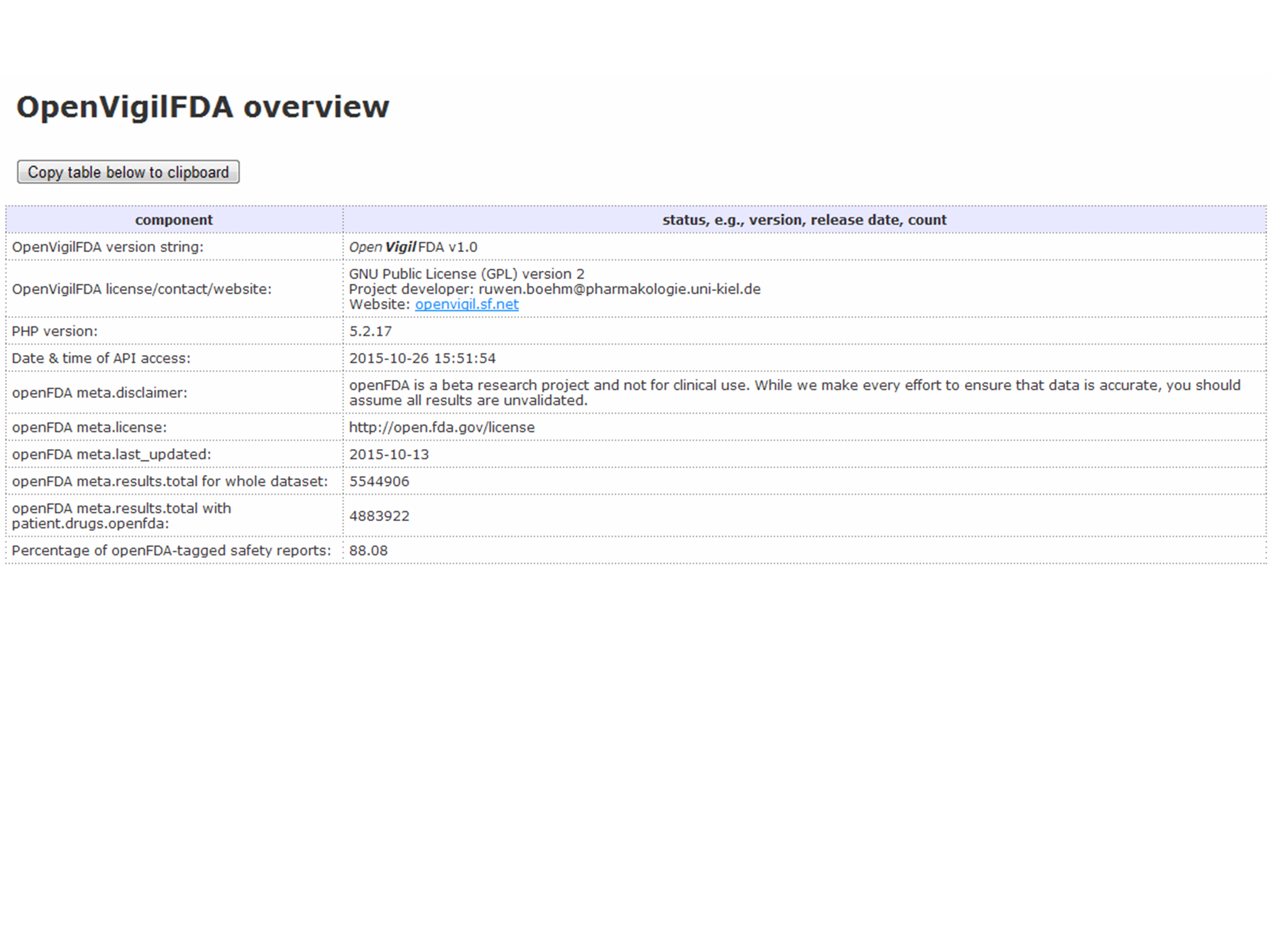

Supplement: S3 Fig — (TIF) [file pone.0157753.s004.TIF]

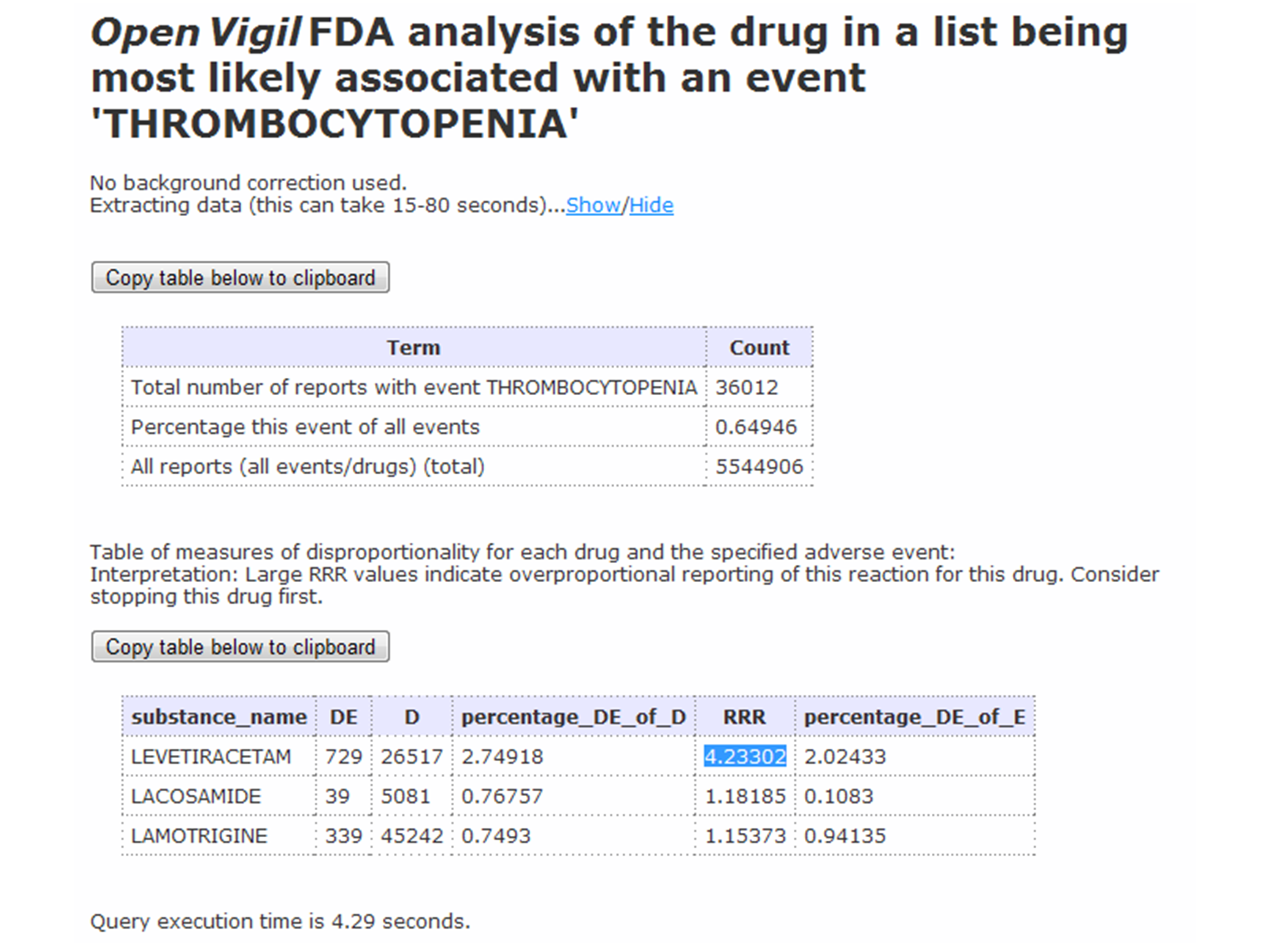

Supplement: S4 Fig — (accessed 2016-02-02). (TIF) [file pone.0157753.s005.TIF]

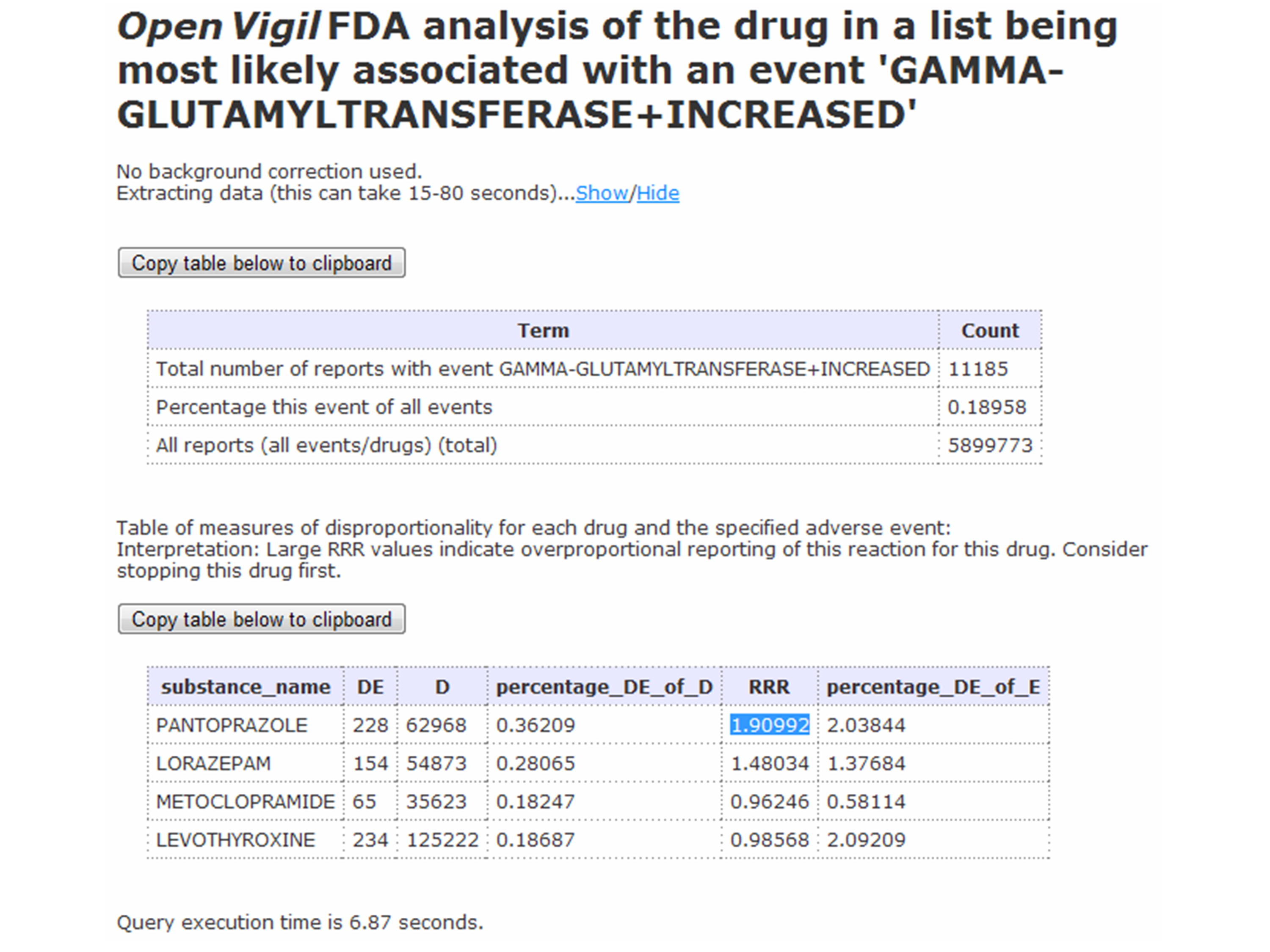

Supplement: S5 Fig — (accessed 2016-01-07). (TIF) [file pone.0157753.s006.TIF]

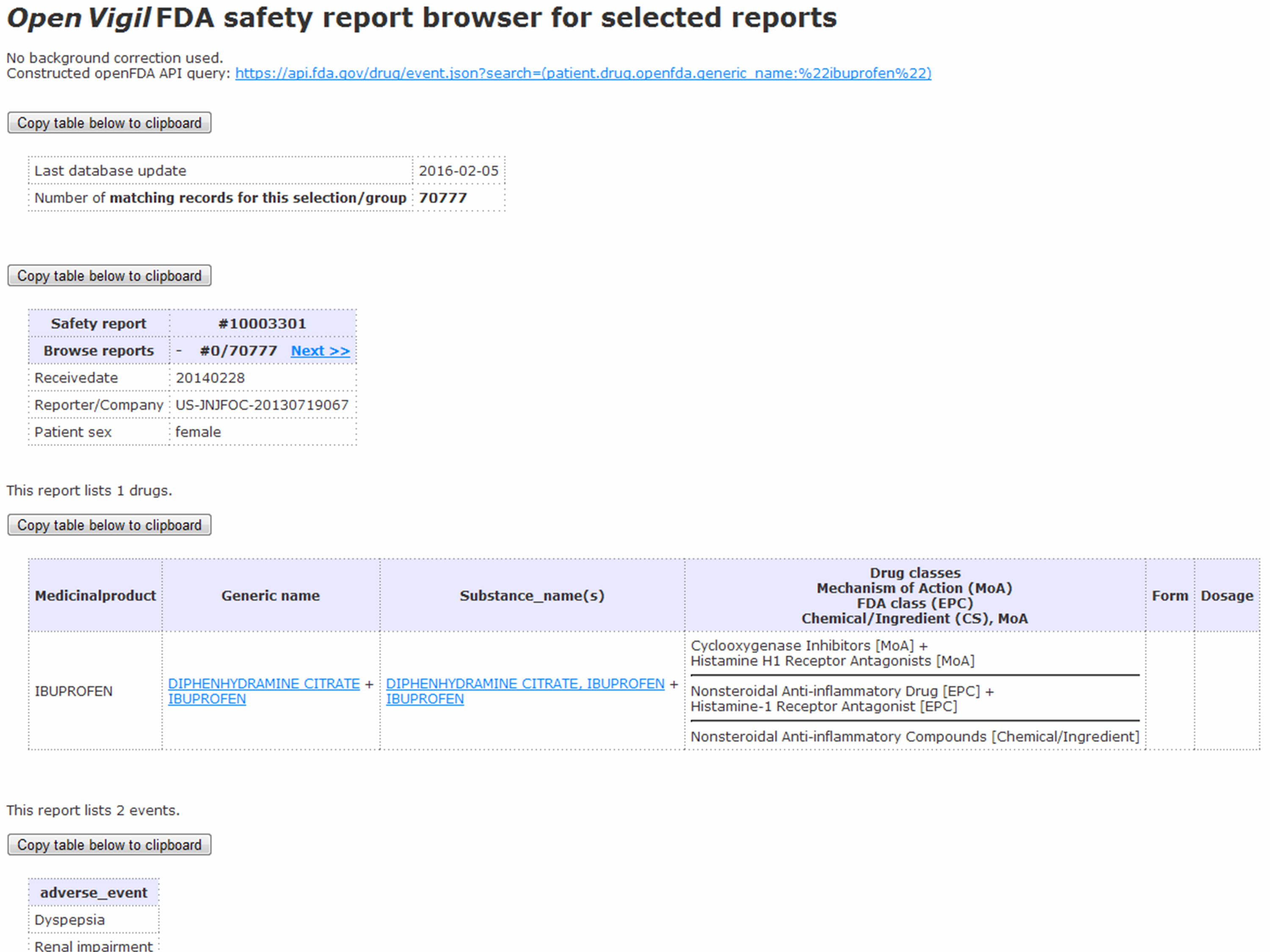

Supplement: S6 Fig — (TIF) [file pone.0157753.s007.TIF]

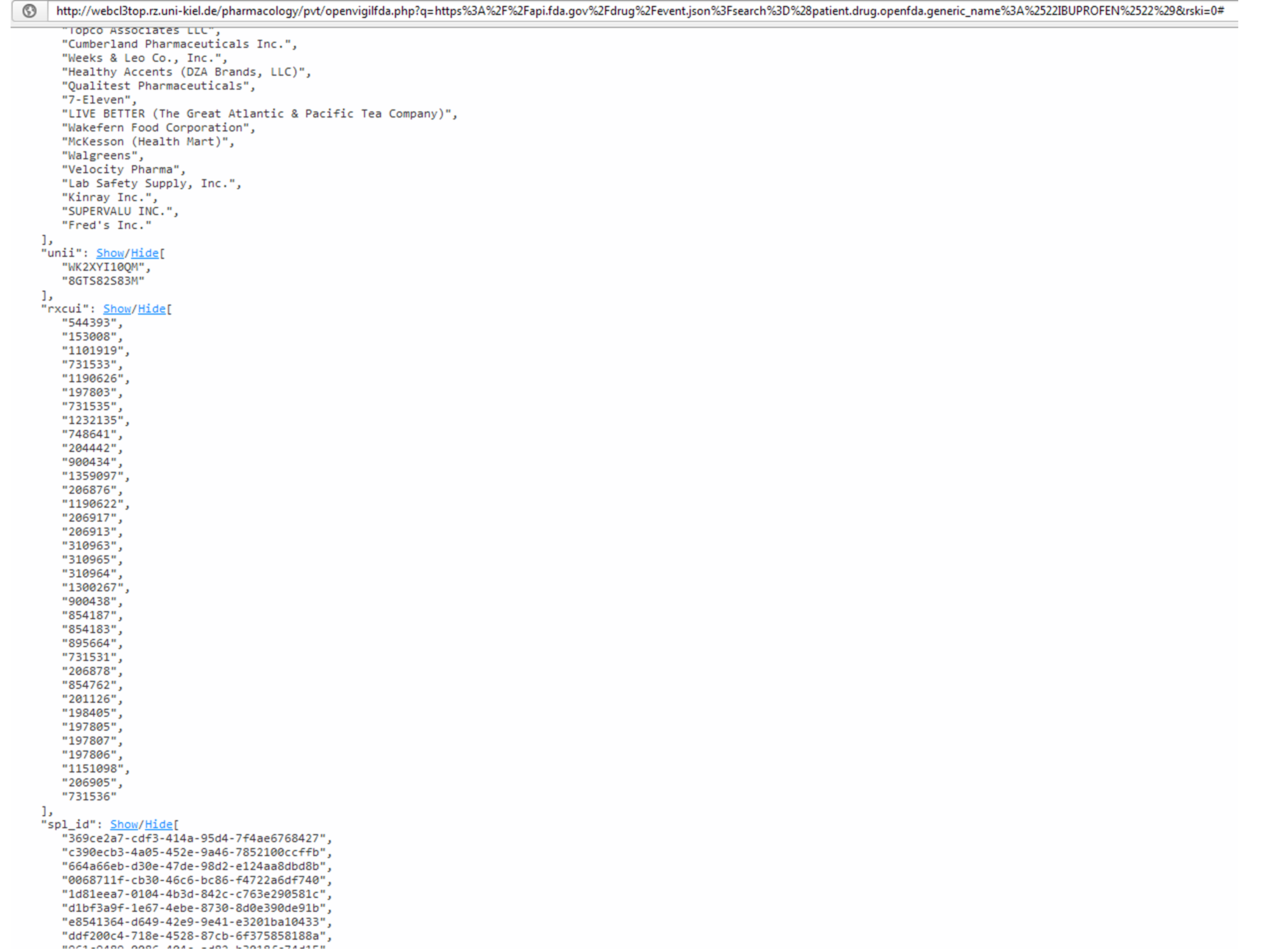

Supplement: S7 Fig — The drug “ibuprofen” is mapped to all possibly matching identifiers (here: manufacturer names, UNII, RXCUI, SPL_ID) resulting in non-involved products and manufacturers being associated with this report. (TIF) [file pone.0157753.s008.TIF]
